# Criminal Abortion

**Published:** 1884-08

**Authors:** 


### CRIMINAL ABORTION.

We learn through our exchanges that, while a medical society has kept free from contamination by expelling a member who was guilty of procuring illicit abortion, conviction by law in another State has failed, on more than one occasion in which the cases have been made out clearly, even to the death of those subjected to such processes. Hence it seems proper at present to direct the attention of those in authority to the Code of Georgia touching this matter, which is as follows:

"Any person who shall wilfully administer to any pregnant woman any medicine, drug or substance, or anything whatever, or shall employ any instrument or means whatever, with intent thereby to produce the miscarriage or abortion of any such woman, unless the same shall have been necessary to preserve the life of such woman, or shall have been advised by two physicians to be necessary for that purpose, shall, upon conviction, be punished as prescribed in section 7310 of the Code."

The penalties are fine, imprisonment, and work in the chain-gang, and any one or more of these punishments may be ordered in the discretion of the judge.

But the old adage of "catching before hanging" comes into play, more especially in this league of a desperate victim of misplaced confidence and an unscrupulous protector of her character in the person of her seducer, with a depraved abortionist.

It is well known by the practicing physicians of Atlanta that many young women in this city, and some who have come from other surrounding States, have been unfortunate, as the phrase goes, in receiving deposits which have not passed current subsequently, and that extraordinary means have been resorted to for the removal of the deposits. How far the bright escutcheon of the medical profession has been tarnished by aiding and abetting this financial transaction, must be decided in the solution of the allegation that every man has his price. That some have borne their crest so loftily as to refuse compliance with the appeals of family influence, and to resist all the temptations of a golden harvest, is in striking contrast with others who have lent a willing hand to screen the victim for the miserable consideration of a few dollars. Those men in the profession who have engaged in this compromise with vicious practices on the part of married and unmarried women, should reflect that, outside of all moral convictions of responsibility, they are encouraging the departure of wives and maidens from the path of duty and virtue, by affording a facility for disposing of the fruits of such illicit intercourse. What is stigmatized as a most nefarious calling in the recognized abortionist, cannot be made less so by the act of a graduate in medicine; and the day must soon come, if it has not already been reached among people of any refinement, when a doctor who accepts this kind of service cannot be received in a respectable house. It is supposed that all such performances are profound secrets between the operator and the patient, yet to-day it is whispered around in Atlanta that sundry would-be-distinguished members of the medical profession have been instrumental in securing the abortion of girls belonging to otherwise reputable families, and that their acts are defended upon the score of protecting the honor of the culprit and of the household. That it has devolved upon some who would have shunned all sorts of relations with such debasement to interfere for the safety of the subjects upon whom abortion has been already initiated, does not implicate them in the criminality of the proceeding; and though a medical man is ordinarily bound to keep inviolate what he learns in the course of his professional associations, so far as his patient is concerned, he is under no such obligation to screen the abortionist, and should, when evidence can be presented, bring any man or woman who may be implicated, to respond for their crimes. Whether in married or unmarried women, the resort to any means of destroying the fruit of conception is infanticide. In a moral, social and legal aspect of the case, an abortionist should be visited by the reprobation of mankind.

It is high time that the honorable members of the profession should assert their claim to consideration apart from those who can be bought up by the gold of the wealthy, or who can be cajoled by family influence, to become accomplices in the crime of illicit abortion. If in the course of professional duty a physician becomes satisfied by good and sufficient evidence that a colleague has knowingly and intentionally produced the miscarriage or abortion of a woman to whom he is subsequently called, there can be no doubt as to the propriety and even duty of exposing this base act of the abortionist.

